# Femtosecond-resolved ablation dynamics of Si in the near field of a small dielectric particle

**DOI:** 10.3762/bjnano.4.59

**Published:** 2013-09-04

**Authors:** Paul Kühler, Daniel Puerto, Mario Mosbacher, Paul Leiderer, Francisco Javier Garcia de Abajo, Jan Siegel, Javier Solis

**Affiliations:** 1 Laser Processing Group (LPG), Instituto de Optica, CSIC, Serrano 121, 28006 Madrid, Spain; 2 Current affiliation: Faculty of Physics, Ludwig-Maximilians-Universität München, Amalienstraße 54, 80799 München, Germany; 3 Current affiliation: Centro de Tecnología Nanofotónica, Universidad Politécnica de Valencia, Edificio 8B, Camino de Vera s/n., 46022 Valencia, Spain; 4 Faculty of Physics, Universität Konstanz, Universitätsstraße 10, 78457 Konstanz, Germany; 5 Instituto de Química Física “Rocasolano”, CSIC, Serrano 119, 28006 Madrid, Spain

**Keywords:** crystalline Si, fs-resolved microscopy, laser ablation, near-field enhancement, ultrafast dynamics

## Abstract

In this work we analyze the ablation dynamics of crystalline Si in the intense near field generated by a small dielectric particle located at the material surface when being irradiated with an infrared femtosecond laser pulse (800 nm, 120 fs). The presence of the particle (7.9 μm diameter) leads to a strong local enhancement (ca. 40 times) of the incoming intensity of the pulse. The transient optical response of the material has been analyzed by means of fs-resolved optical microscopy in reflection configuration over a time span from 0.1 ps to about 1 ns. Characteristic phenomena like electron plasma formation, ultrafast melting and ablation, along with their characteristic time scales are observed in the region surrounding the particle. The use of a time resolved imaging technique allows us recording simultaneously the material response at *ordinary* and *large* peak power densities enabling a direct comparison between both scenarios. The time resolved images of near field exposed regions are consistent with a remarkable temporal shift of the ablation onset which occurs in the sub-picosend regime, from about 500 to 800 fs after excitation.

## Introduction

The term “near ﬁeld optics” is used to describe the phenomena associated to non-propagating and highly localized electromagnetic fields and their interaction with matter. Optical near ﬁelds (ONF’s) can be generated in the vicinity of metal or dielectric nanoparticles, leading to physical properties that are drastically different from their free-propagating counterparts [1]. Evanescent waves, for instance, are not purely transverse and ONF’s have the ability to localize electromagnetic energy to length scales well below the diffraction limit [2]. Moreover, the localization of electromagnetic energy in the vicinity of a small metallic or dielectric scattering particle leads to a local increase of the intensity of an incoming electric field. As a consequence, the electric field can be enhanced by several orders of magnitude which is particularly useful for exciting optical non-linearities (Kerr, Raman, … [3–4]) in the vicinity of metal particles.

**Figure 1:**
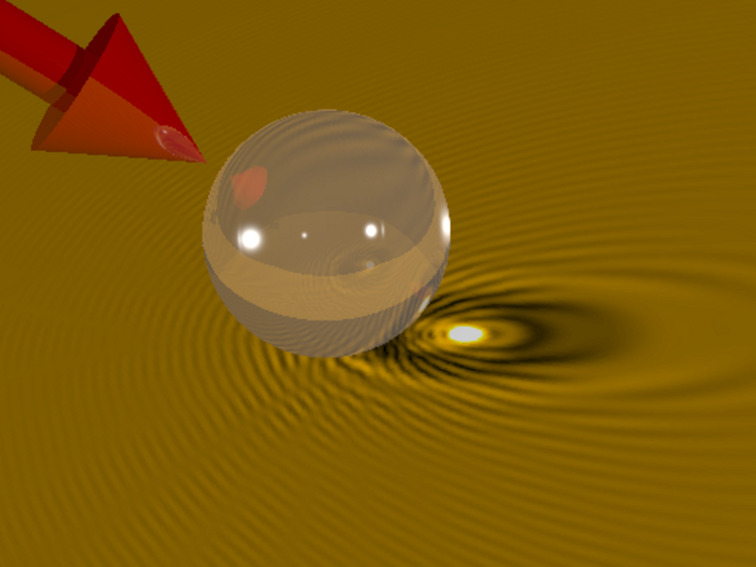
Schematic view of the spatial intensity distribution at the surface of a crystalline Si substrate in the vicinity of a 7.9 μm-diameter SiO_2_ spherical particle illuminated at oblique incidence by a laser with 800 nm wavelength. The bright spot corresponds to the enhancement of the incoming field. The fine fringe structure is caused by interference of the incoming laser beam, light reflected at the substrate, and light scattered by the particle.

For dielectric particles, Mie scattering can similarly lead to strong field enhancement effects [5–6] which have found increasing applications in nano-structuring [7–8] and materials nano-fabrication [9–10]. As an example, Figure 1 shows a schematic 3D representation of the calculated intensity distribution at the surface of a crystalline Si wafer in the vicinity of a 7.9 μm-diameter SiO_2_ spherical particle, illuminated at oblique incidence by a laser with a wavelength of 800 nm. The intensity distribution shows a bright spot corresponding to an enhancement factor of the order of 40, evidencing that in a first approach the particle (with a Mie parameter *ka* ≈ 40, where *a* is the particle radius and *k* the wave vector of the scattered light) behaves like a spherical lens. A closer look at the spatial distribution of intensities reveals a fine structure with characteristic maxima and minima that cannot be predicted by a simple geometrical model and that depends on the complex interference of the incoming laser beam, the light reflected at the substrate surface, and the light scattered by the particle [11]. If we consider an excitation pulse of 100 femtoseconds (fs) duration reaching the surface with a homogenous fluence of 1 J/cm^2^, the local fluence at the maximum of the near field distribution would reach 40 J/cm^2^ or 400 TW/cm^2^. To reach such a peak power density using a short focal length lens would be difficult, among other reasons, due to the fact that we would exceed by large the critical power for self-focusing and induce dielectric breakdown of air, leading to pulse distortion. ONF’s enable thus to investigate laser-matter interaction processes at large power densities, difficult to achieve using conventional means.

In this work we have analyzed the ablation dynamics of crystalline silicon (c-Si) under the intense near field caused by a small dielectric sphere located at the material surface when being irradiated with a 120 fs long 800 nm wavelength laser pulse. In the study, we have reached locally enhanced fluences up to 35 J/cm^2^ (≈300 TW/cm^2^) (for comparison the fluence threshold for surface ablation is ≈0.5 J/cm^2^ [12]) and used fs-resolved microscopy in order to access time scales associated to characteristic events, like plasma formation, ultrafast melting or material ablation. The material behavior in the near field region is consistent with a pronounced temporal shift of the characteristic interaction events with respect to that observed at the much lower fluences present outside the ONF region (up to 0.91 J/cm^2^). Interestingly, the lift-up time of the particle upon ablation of the near field exposed region is beyond 20 ns, which is the maximum temporal window accessible with our setup.

## Experimental

### Sample preparation

Spherical SiO_2_ particles with 7.9 μm in diameter (Bangs Laboratories, Inc., nominal polydispersity 2%) stored in iso-propanol, were deposited on a Si(001)-wafer by means of spin coating. Using this technique it is feasible to control the particle density as well as it features prevalent particle isolation in the resulting distribution, with a particle separation much larger than the size of the irradiation beam.

In order to image the expected near field structure upon surface illumination a sample consisting of 40-nm-thick, face-centered-cubic (fcc) polycrystalline Ge_2_Sb_2_Te_5_ (GST) on a Si(001) substrate covered by a 10-nm-thick amorphous SiO_2_ film was used. Further details regarding the preparation procedure and optical properties of the different substrates and particles used, as well as near field imaging using GST films can be found elsewhere [11].

### Femtosecond laser irradiation and time-resolved microscopy

**Figure 2:**
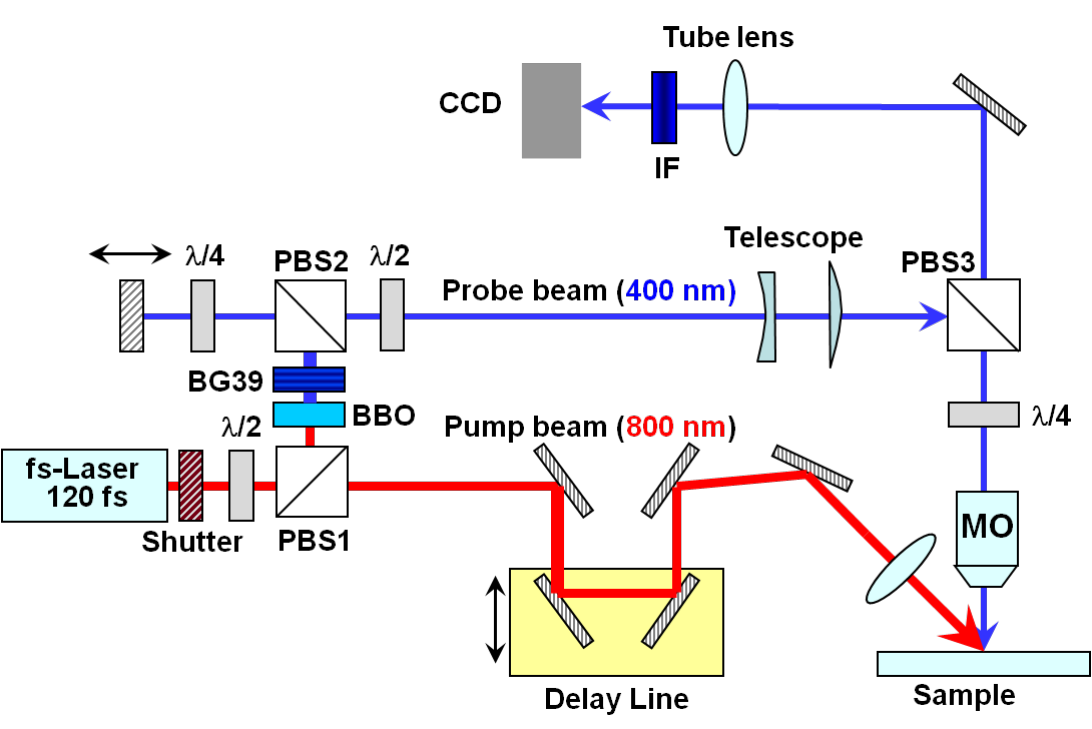
Scheme of the experimental set-up used for sample irradiation and fs-resolved microscopy. (IF) interferential band-pass filter centered at 400 nm, PBS (polarizing beam-splitter cubes), (BBO) beta barium borate frequency doubling crystal, (MO) microscope objective, (λ/2, λ/4) waveplates, (BG39) high frequency band-pass filter.

Figure 2 shows a scheme of the set-up used for irradiating the samples and acquiring the fs-resolved surface reflectivity images. The pump-probe microscope uses a single 120 fs pump pulse at 800 nm, which was selected by using an electro-mechanical shutter. The pump beam (s-polarized) is focused at an angle of incidence of 54° onto the sample surface to a (elliptical) size of 106.6 μm × 62.2 μm (1/*e*^2^ intensity diameter). An optical delay line allows controlling the relative times of arrival to the surface of the pump and probe pulse. The combination of a λ/2-wave-plate at 800 nm and a polarizing beam splitter (PBS1) enables extracting a small fraction of the pump beam which is used for generating the frequency-doubled probe beam by means of a β-BaB_2_O_4_ (BBO) crystal and a BG39 optical short pass filter. A coarse delay line for the probe pulses is then configured by means of another polarizing beam splitter PBS2 and a λ/4-wave-plate at 400 nm. The region irradiated by the pump pulse is imaged (for a given delay) by using the 400 nm probe pulse as illumination source, combined with a telescope, a long working distance microscope objective (MO, 20×, NA = 0.42), and a tube lens. A polarizing cube beam splitter (PBS3) is used to steer the probe beam in order to illuminate the surface at normal incidence while a λ/4-wave-plate at 400 nm ensures that the light reflected from the sample passes back through PBS3 towards the imaging system. A narrow band-pass filter centered at 400 nm blocks the pump pulse scatter and plasma emission while the probe light reaches the (12-bit) CCD, which records the images. The temporal resolution of the system (mostly limited by group velocity dispersion of the 400 nm probe beam in the PBS3 and the MO) is about 300 fs. Further details about the irradiation and imaging setup can be found elsewhere [13–14]. It is worth noting, though, that the spatial Gaussian intensity profile of the irradiation beam results in a position-dependent fluence distribution. As a consequence, a reflectivity image for a given delay contains not only spatial information but also information about the fluence dependence of the process.

The sample is mounted on a computer controlled micrometer-adjustable three-axis translation stage. Since irradiation leads, in most cases, to permanent structural modifications of the surface, each region containing a particle is irradiated only once for a given delay between the pump and probe pulses. Irradiations under the same conditions are similarly acquired in particle-free regions for comparison purposes.

### Image processing

During the image acquisition process, three different images are acquired for a given delay. A first image, blocking the pump beam, is used for normalization purposes. This enables normalizing the transient reflectivity changes recorded in the second image, acquired for a given delay with the pump beam unblocked. A third image of the already irradiated surface is acquired and normalized as indicated in order to observe the state of the surface after irradiation. This image is acquired typically a few seconds after irradiating the surface. When a particle is present in the irradiated regions surface this normalization procedure also helps in reducing the strong scattering caused by the particle. As a consequence, in the time-resolved images, the particle is barely visible, although small beam pointing fluctuations can render an image normalization process not completely effective.

#### Near field distribution simulations

The spatial intensity distribution produced by the particle at the substrate plane upon illumination has been calculated by a rigorous solution of Maxwell’s equations for a sphere sitting on a planar substrate, using the method fully elaborated in [11], based on the methods described in [15].

## Results and Discussion

### Structural transformation dynamics of the particle-free Si surface

**Figure 3:**
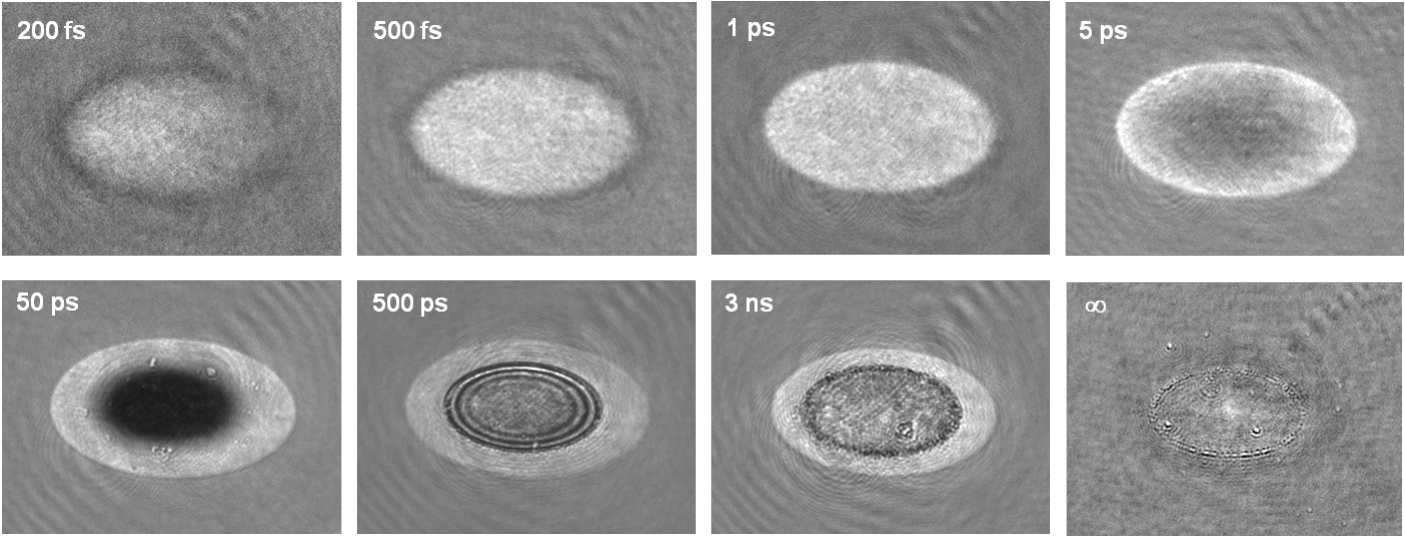
Surface reflectivity images of a particle-free c-Si surface exposed to a 120 fs laser pulse for different time delays between the excitation and illumination pulses. The peak fluence is 0.91 J/cm^2^. The frame size is 93 × 69 μm^2^. The brightness and contrast of each image have been adjusted to improve the visibility of the main features.

**Figure 4:**
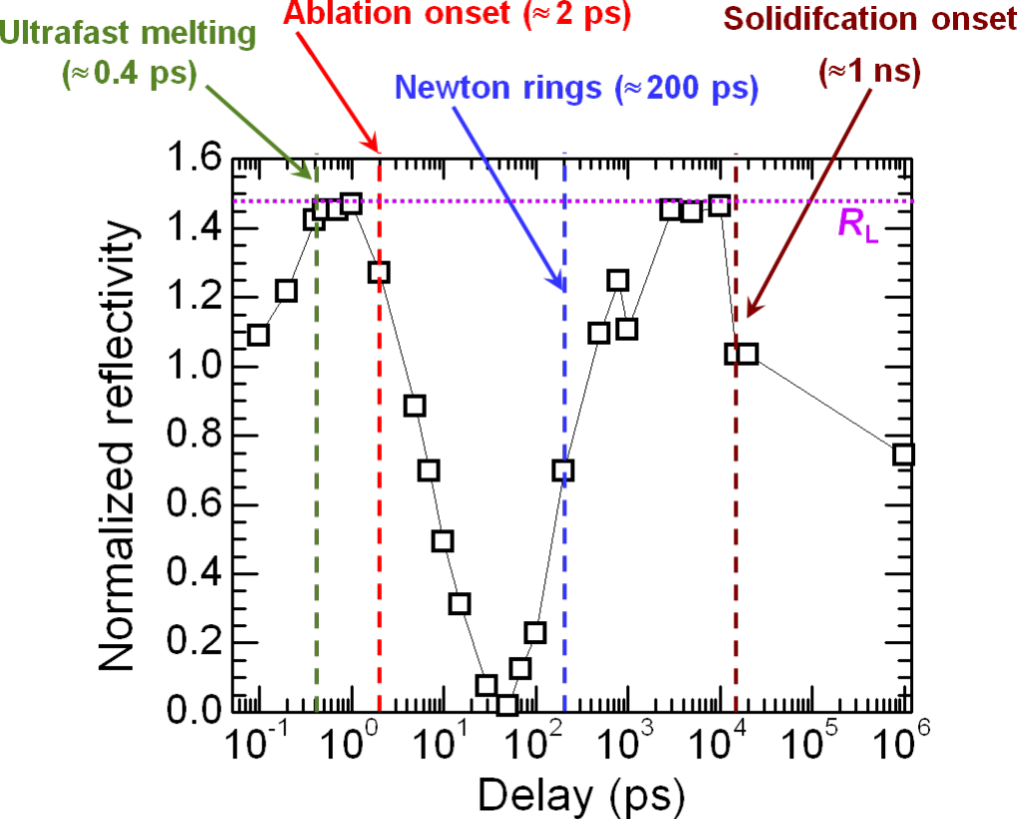
Normalized reflectivity as a function of delay measured for a particle-free, c-Si surface, at the center of the irradiated region for a peak fluence of 0.91 J/cm^2^. The point at 10^6^ ps corresponds to an actual delay of several seconds (denoted as ∞ in Figure 3). The onset of the most characteristic events is indicated by arrows. R_L_ indicates the relative reflectivity at 400 nm (1.48) of an optically thick molten liquid layer. The line is a guide to the eye.

In order to compare the temporal evolution of the material in a particle’s near field to that of a particle-free surface we have performed a dedicated experiment on bare Si. Figure 3 shows several images of the evolution of the reflectivity of a particle-free, c-Si surface upon exposure to a 120 fs laser pulse with a peak fluence of 0.91 J/cm^2^. With a reflectivity of 0.52 of the surface at 54° for s-polarized light, this corresponds to an absorbed fluence of 0.44 J/cm^2^. Since the melting and ablation dynamics of c-Si upon fs-laser excitation has already been analyzed in detail [16–17] we will briefly describe only the main features of the process. To facilitate the description we have included in Figure 4 the evolution of the surface reflectivity measured at the center of the irradiated region, i.e., at the position of maximum fluence.

The image at 200 fs delay shows the formation of a bright region in the laser irradiated zone due to the formation of a dense free-electron plasma. The decrease of the plasma reflectivity along the horizontal axis from the left to the right is the consequence of the irradiation angle which makes the pulse to reach the surface with an increasing delay. The difference in the “landing time” of the irradiation pulse over the 1/*e*^2^ diameter of the beam is about 200 fs. At longer delays, the reflectivity of the irradiated region increases and reaches a saturation value very close to the reflectivity expected for molten Si at approximately 400 fs (see Figure 4). It is worth noting that this saturation value is “fluence independent”, i.e., it is the same over the whole bright region showing a sharp threshold. This points to the fact that the material has undergone the so called *ultrafast* or *non-thermal* melting process, a structural transition due to the instability of the crystal lattice in the presence of a dense electron–hole plasma (≈10^22^ cm^−3^, 10% of the whole valence population) [18]. Approximately 1 ps after excitation, the highly excited material relaxes to *thermal* liquid-Si.

The reflectivity at the center of the irradiated zone starts to decrease afterwards (see in Figure 3 the image at 5 ps delay) showing a characteristic dark zone which will be observable for delays up to 100 ps. This reflectivity decrease is indicative of the ablation of the surface. The emission of ions and neutral species in the ablating region leads to a strong absorption of the illumination probe beam. The ablation onset occurs at about 2 ps, although strong ablation effects (a reflectivity decrease below half of the maximum) are observed later, at about 10 ps (see Figure 4). The dark zone is surrounded by a brighter ring corresponding to liquid Si. The reflectivity in the center reaches its minimum in 50 ps and starts to recover showing a characteristic ring structure (Newton rings) over a time window of 200–1000 ps (see in Figure 3 the image at 500 ps delay). The observation of Newton rings is due the formation of a transparent but dense ablating layer with a sharp interface [18]. The rings disappear after a few nanoseconds, as can be seen in the image for a 3 ns delay in Figure 3. At this delay it can also be noticed that the extension of the molten region surrounding the ablated crater is shrinking as a consequence of solidification. After ca. 20 ns no further structural changes are observed at the surface, which features an ablated crater (see in Figure 3 the image at ∞, corresponding to a few seconds delay). The thresholds for thermal melting and ablation deduced from the images are 0.26 J/cm^2^ and 0.54 J/cm^2^, respectively (corresponding to absorbed fluences of about 0.13 J/cm^2^ and about 0.26 J/cm^2^).

### Structural transformation dynamics at the near field excited Si surface

**Figure 5:**
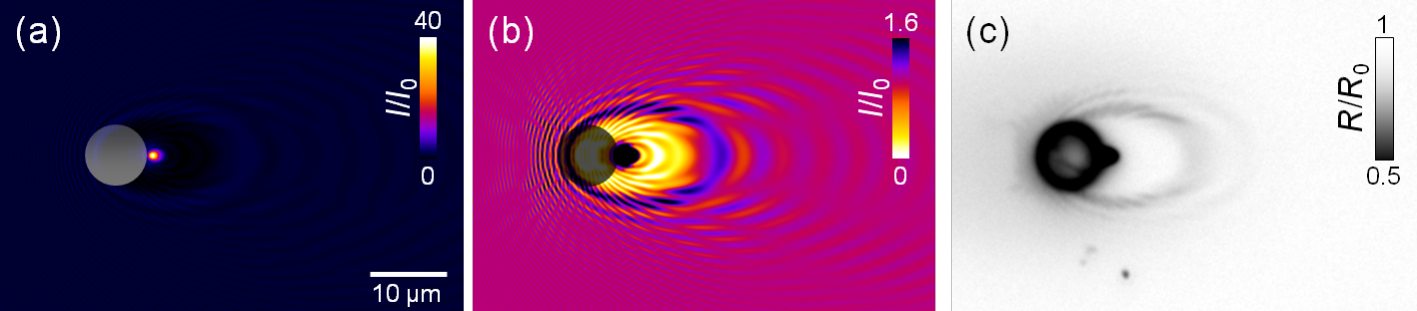
(a) Calculated near field distribution at the surface of a Si substrate for a SiO_2_ dielectric particle of 7.9 μm diameter homogenously illuminated at a wavelength of 800 nm and at an angle of 54°. The position of the particle is shown as a semitransparent circle. The spatial intensity distribution has been normalized to the incoming beam intensity (*I*_0_). (b) Same as (a) but re-scaling the color scale to a maximum enhancement factor (*I*/*I*_0_) of 1.6. As a consequence the intensity distribution at the region of the maximum intensity of (a) is saturated (black color). (c) Optical reflectivity micrograph of the surface of a GST film on c-Si after exposure to a 12.3 mJ/cm^3^ peak fluence pulse at the same angle of incidence.

In order to gain a better insight of the expected features when irradiating a particle placed on the Si surface, we have calculated the expected near field intensity distribution as shown in Figure 5a. Nearby the dielectric particle a very small spot where the incoming intensity is enhanced by a factor of about 40 can be observed. A closer look at the image indicates the presence of a fine structure showing characteristic interference maxima and minima. In order to better appreciate these details, in Figure 5b, the same intensity distribution has been plotted with a saturated intensity scale (displaying only up to 1.6) and inverting colors for clarity (the region of maximum enhancement now looks black). A complex intensity distribution is seen around the particle, where the observed ring period is larger in the beam propagation direction (forward) than in the backscattered one. Regions with local intensity values above and below that of the incoming illumination beam can be distinguished. The period of the observed intensity distribution along the surface projection of the beam propagation axis far from the particle corresponds to λ_inc_/(1 ± sinθ) (i.e., ≈0.4 and ≈4.0 μm, respectively, for the backward and forward directions, where θ is the angle of incidence) as expected for the interference of a spherical wave with a scattering source [11]. For comparison, in Figure 5c we have included an optical micrograph (in reflection) obtained upon illumination of a GST film with a 120 fs laser pulse with a peak fluence of 12.3 mJ/cm^2^, at the same angle of incidence. As shown in [11], GST (see above) has been successfully used to image and quantitatively analyze the spatial near field intensity distribution generated by a particle on a surface by imprinting a corresponding pattern through local amorphization.

We can see that the intensity modulation associated to the near field is replicated by a periodic variation of the reflectivity of the illuminated material, which is formed by alternating amorphous and crystalline regions depending on whether the local fluence is above or below the melting threshold of polycrystalline GST. We have checked that the calculated spatial distribution of the near field is essentially the same for both Si and GST substrates.

The comparison between the imprinted and calculated near field distribution shows an excellent agreement which allows us to conclude that our calculation of the maximum field enhancement expected (≈40) on the Si substrate for the particle size and irradiation geometry considered is quite accurate. When a pulsed laser of moderate fluence illuminates the surface, the local fluence close to the particle can thus reach easily a value above the ablation threshold of the surface. This leads to the removal of the particle, which is ejected from the surface due to the pressure generated by the expanding plasma, as shown in several “dry” laser cleaning experiments with short laser pulses [5].

**Figure 6:**
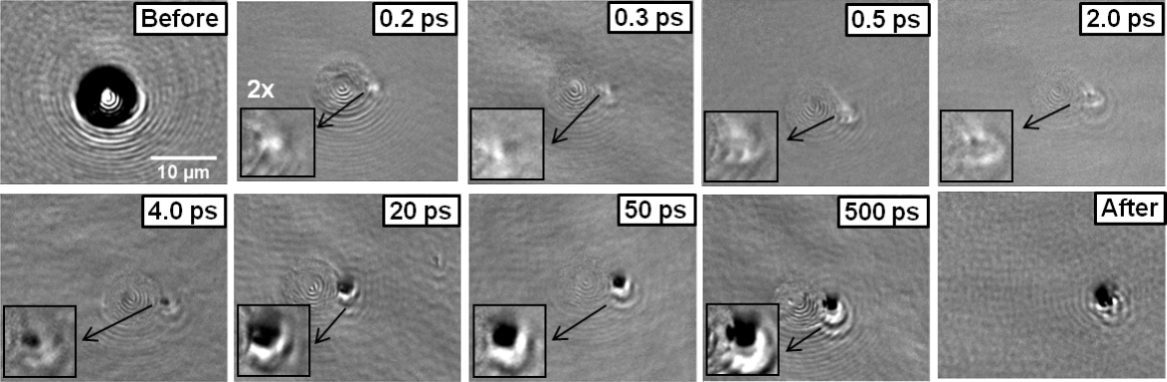
Surface reflectivity images at the particle location for different time delays, upon exposure to a 120 fs-laser pulse with a peak fluence of 0.11 J/cm^2^ reaching the surface at 54°. With the exception of the images corresponding to the surface before and after the arrival of the laser pulse, the images have been normalized to an image obtained blocking the pump pulse in order to minimize the scattering effects caused by the 7.9 μm-diameter particle. The delay is indicated in each image. A zoom (2×) has been applied to the relevant part of the image and pasted as an inset to facilitate the description in the text.

Figure 6 shows the reflectivity evolution at the particle location for illumination with a peak fluence of 0.11 J/cm^2^, which is below the melting threshold of the surface (0.26 J/cm^2^) but above the threshold for particle removal (0.09 J/cm^2^). Images corresponding to the surface before and after the arrival of the illumination pulse to the surface are included for reference. The image “before” shows the particle as a round scattering, dark contrasted circle. In order to reduce scattering artifacts in the time-resolved images they have been normalized (except in the one obtained “after”, with a delay of several seconds, as above indicated) by dividing them by an image obtained blocking the pump laser. As a consequence, the position of the particle can be appreciated as a set of concentric rings with a smooth grey contrast.

In the following we will compare the behavior observed in the images of Figure 6 to the behavior of the particle-free surface in Figure 3. In this latter case the surface experiences a peak fluence of 0.91 J/cm^2^ while the region of maximum field enhancement for the particle corresponds to ca. 4 J/cm^2^. For the comparison of both sets of images we have to consider that the spatial resolution of the imaging system is limited (≈1 μm). As a consequence, any spatial feature, even with a strong reflectivity contrast, will appear blurred and feature a reduced contrast if its size is below the resolution limit. As a consequence, it is not possible to provide a plot with the quantitative temporal evolution of the reflectivity similar to that shown in Figure 6.

As we can see in Figure 6, already for a delay of 200 fs we can observe the formation of a bright spot located adjacent to the particle position (coinciding with the bright region in Figure 5a where maximum field enhancement is induced) which evidences the formation of a dense electron plasma. The next image, at 300 fs, shows that both the intensity and size of the bright region at the edge of the particle are reduced. For further delays (0.5–2 ps), the images show a very similar appearance, with a small ellipse (see the insets with a 2× zoom of the relevant region) with a bright perimeter that scatters the probe beam light. We know from the behavior of the particle-free surface that the ablation onset occurs (for much lower local fluences) for delays as short as 2 ps (see Figure 4). The similarity of the images in the delay interval from 500 fs up to 2 ps thus strongly suggest that the formation of the ellipse with bright borders (lower reflectivity at the center) might be indicative of the onset of ablation at large local fluences (4 J/cm^2^) already after less than 1 ps (500–800 fs, considering the time resolution of the experimental setup). When considering this temporal delay, (500–800 fs) it must be emphasized that the ablation process (thermal) requires the transfer of energy from excited carriers to the lattice via e-ph scattering. The ablation onset cannot therefore be temporally shifted to times shorter than the characteristic e-phonon scattering time.

Different carrier density dependent relaxation mechanisms have been proposed to explain extremely large collision frequencies (>2 × 10^15^ s^−1^) in c-Si for carrier densities above 10^22^ cm^−3^ (see [16] and references quoted therein). For what concerns the e-phonon scattering time, a carrier relaxation lifetime of only 240 fs, involving ultrafast lattice heating, has recently been reported for Si using two-photon photoemission spectroscopy [19]. Even assuming the fact that ablation is in itself a thermal process and considered as slow [20], the temporal border between electronic excitation alone and lattice heating is somewhat diffuse. The fact that thermal melting is already observed at a 2 ps delay at lower peak fluences in the particle-free surface points to a shorter (<1 ps) ablation onset for the much larger fluence in the near field exposed region. Indeed, at a 4 ps delay, the formerly bright region (at 200 fs) turns dark, indicating that strong ablation is already taking place. A comparison of the images at 5 ps and 50 ps delays for the particle-free surface (Figure 3) and the image for 4 ps in Figure 6 clearly shows that the onset of strong ablation has clearly shifted to shorter times in the near field region. These observations are consistent with an ablation onset occurring 500–800 fs after excitation in the near field region, as indicated in the previous paragraph. This delay, of the order of twice the e-ph scattering time, is close to the temporal limit for which ablation can be first observed after excitation.

Interestingly, unlike the case of the particle-free surface, in the time lapse from 4 ps to 20 ps the size of the ablating region expands (see 2× zoom region for both delays in Figure 6). This is indicative of collateral effects of the ablating region, initially confined to a very small region, unlike in Figure 3. The maximum contrast of the ablating dark region is reached within about 50 ps. Still, in the 50–500 ps interval, the lateral dimensions of the black spot and its contrast remain essentially unchanged. It is therefore not possible to assess when the ablation process terminates, as the observed contrast in both images may be caused by plasma absorption or by the formation of a hole of several tens of nm depth, as seen in the image labeled “After” in Figure 6. We can conclude though, from images for a delay of 20 ns (not shown) where the particle is still observed that the particle lift-up is largely delayed. A crude estimation of the maximum velocity of the particle can be obtained by assuming the particle travels a distance of the order of the Rayleigth range of the imaging lens used, ≈1 μm at 400 nm (equivalent to half of its depth of focus, DOF) in time lapse of the order of more than 20 nanoseconds. This leads to a maximum value below 50 m/s, or an acceleration below roughly 10^9^ m/s^2^ (10^8^ g_0_), equivalent to a force of ≈0.5 × 10^−3^ N. Obviously the relative slowness of particle lift up process is related to the large mass of the particle when compared to that of light ablation products (neutrals, ions, …).

**Figure 7:**
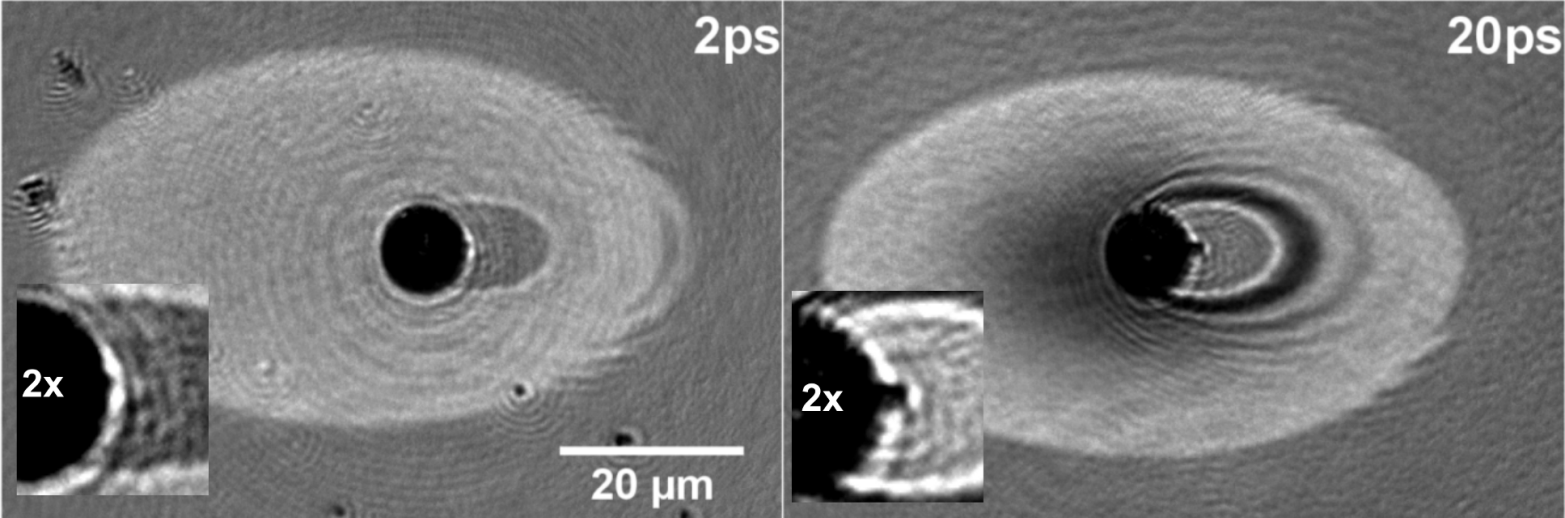
Surface reflectivity images at the particle location for two different delays (indicated), upon exposure to a 120 fs-laser pulse with a peak fluence of 0.87 J/cm^2^ reaching the surface at 54º. A zoom (2×) has been applied to the relevant part of the image and pasted as an inset to facilitate the description in the text.

In order to analyze the material response at even higher local fluences and confirm the apparent temporal shift of the ablation process above discussed, experiments with a peak fluence of 0.87 J/cm^2^ (corresponding to a field-enhanced fluence 34.8 J/cm^2^) were also performed. Figure 7 includes two relevant images corresponding to delays of 2 and 20 ps. In this case, the images have not been normalized to minimize the noise scattering effect associated to the particle which appears as a black sphere at the center. The peak fluence used is above the ablation threshold and comparable to the one corresponding to the images in Figure 3 (0.91 J/cm^2^). We can see how a molten silicon layer is present at the surface (see above description of Figure 3) for a delay of 2 ps. A close-up look to the region nearby the particle (see the 2× zoomed inset) shows though a tiny black dot adjacent to the position of the sphere which evidences that for this delay the focus region is already undergoing strong ablation. This observation is consistent with the above discussed temporal shift of the ablation process into the sub-picosecond range. The effect of the field enhancement can actually also be seen at the right edge of the molten region where part of the fine structure of the near field and far field scatter distribution is visible in form of a structured distribution of molten material. Indeed, the light scattered by the particle in the forward direction induces melting outside the region where the pump beam is concentrated.

The region where maximum field enhancement is achieved is more evident in the image for 20 ps, as the dimensions of the strongly ablating spot are now larger (see also the 2× zoomed inset). The “tiny black dot” observed in the 2 ps image is now a nearly elliptically shaped region undergoing, as expected, strong ablation. The effect of the near field, apart from the region of maximum field enhancement, is also visible as a characteristic ring pattern. It shows bright regions where destructive interference has inhibited the onset of ablation and dark regions where constructive interference drives the material into the strong ablation regime. This is evident when comparing the overall appearance of the image to that shown in Figure 5b. It is interesting to notice that there are no completely “shaded” regions and material melting occurs also within the shadow region of the sphere. This is an effect of the near field and fully consistent with the calculated intensity distribution shown in Figure 5b.

## Conclusion

In this work we have analyzed the ablation dynamics of crystalline Si in the near field region of a dielectric particle upon illumination with a 120 fs laser pulses at 800 nm. For this purpose we have used fs-resolved microscopy to compare the behavior of regions excited inside and outside the near field region generated by a 7.9 μm-diameter SiO_2_-sphere for different time delays where characteristic interaction events (plasma formation, ultrafast and thermal melting and ablation) can be observed. The recorded time resolved images are consistent with a temporal shift of both the ablation onset and the strong ablation regime to shorter delays. For the ablation onset, enhanced local intensities of the order of 4 J/cm^2^ lead to an ablation onset in the sub-picosecond range (500–800 fs) while for local intensities of the order of 35 J/cm^2^ strong ablation can be observed for delays as short as 2 ps. These time scales are likely in the limit of observable ablation (thermal) processes as the delays for which ablation is first observable reach values close to the e-phonon scattering time in crystalline Si [19]. Images of the surface obtained for long delays indicate that in spite of the large local fluences achieved, particle lift up, caused by the ablation of the underneath material is a relatively slow process. The time required for the particle to traverse a distance of the order of the Rayleigh range of the imaging optics used is beyond 20 ns which indicates that maximum particle accelerations achievable are of the order, or below, 10^9^ m/s^2^ (at least for particles with a Mie parameter well above one).

The results achieved also show that near field effects can be used to analyze ultrafast laser-matter interaction phenomena at large fluences avoiding, in a relatively simple manner, undesirable side effects of large peak power interactions, like self-focusing and dielectric breakdown in air. Time-resolved microscopy provides then extremely valuable information of the interaction dynamics.
